# Anxiolytic effects of Formononetin in an inflammatory pain mouse model

**DOI:** 10.1186/s13041-019-0453-4

**Published:** 2019-04-08

**Authors:** Xin-shang Wang, Shao-yu Guan, An Liu, Jiao Yue, Li-ning Hu, Kun Zhang, Liu-kun Yang, Liang Lu, Zhen Tian, Ming-gao Zhao, Shui-bing Liu

**Affiliations:** 1 Department of Pharmacology, School of Pharmacy, and Precision Pharmacy & Drug Development Center, Department of Pharmacy, Tangdu Hospital, Fourth Military Medical University, Xi’an, 710032 China; 2 The 154th Central Hospital of PLA, Xinyang, 464000 China

**Keywords:** Inflammation, Chronic pain, Anxiety, Formononetin, Amygdala, NMDA

## Abstract

Chronic pain is commonly accompanied with anxiety disorder, which complicates treatment. In this study, we investigated the analgesic and anxiolytic effects of Formononetin (FMNT), an active component of traditional Chinese medicine red clover (*Trifolium pratense* L.) that is capable of protecting neurons from N-methyl-D-aspartate (NMDA)-evoked excitotoxic injury, on mice suffering from complete Freund’s adjuvant (CFA)-induced chronic inflammatory pain. The results show that FMNT administration significantly reduces anxiety-like behavior but does not affect the nociceptive threshold in CFA-injected mice. The treatment reverses the upregulation of NMDA, GluA1, and GABA_A_ receptors, as well as PSD95 and CREB in the basolateral amygdala (BLA). The effects of FMNT on NMDA receptors and CREB binding protein (CBP) were further confirmed by the potential structure combination between these compounds, which was analyzed by in silico docking technology. FMNT also inhibits the activation of the NF-κB signaling pathway and microglia in the BLA of mice suffering from chronic inflammatory pain. Therefore, the anxiolytic effects of FMNT are partially due to the attenuation of inflammation and neuronal hyperexcitability through the inhibition of NMDA receptor and CBP in the BLA.

**Electronic supplementary material:**

The online version of this article (10.1186/s13041-019-0453-4) contains supplementary material, which is available to authorized users.

## Introduction

Patients suffering from chronic pain often have emotional comorbidities that affect mood, sleep, activity, and cognition. Accordingly, the prevalence of anxiety disorders among patients with chronic pain ranges from 20 to 40%, compared to 7–18% in the general population [1, 2]. Epidemiological studies have reported that the pervasiveness of pain in subjects with anxiety or depression, and that of anxiety or depression in subjects with pain, are higher than in the cohort with either condition alone [3–5]. Opioids are the most effective treatment for pain. However, the incidence of anxiety among opioid-treated chronic pain patients is 48.4% [6]. Thus, an effective treatment of chronic pain requires a combination of analgesics as well as anxiolytics [7].

Formononetin (FMNT), one of the main active components in *Trifolium pratense* L. medicinal plant, is described as a typical phytoestrogen [8]. It is involved in the fracture-repair process, as evidenced by its role in enhancing bone regeneration in a mouse model of cortical bone defect [9]. Moreover, it has hypolipidemic properties and free radical scavenging activity that prevent the formation of lipid peroxidation [10, 11]. FMNT is also reported to have a neuroprotective effect against oxidative stress and excitotoxicity instigated by hydrogen peroxide and L-glutamate [12, 13]. In a previous study, we showed that FMNT protected neurons from N-methyl-D-asparate (NMDA)-induced apoptosis [14]. Nevertheless, the effects of FMNT on analgesia and antianxiety are not well known.

The amygdala, a critical region associated with emotion and motivation, consists of several anatomically and functionally distinct nuclei, including the lateral (LA) and basolateral (BLA) nuclei, as well as the central nucleus (CeA) [15]. Among the subnuclei of the amygdala, the BLA bi-directionally communicates with brain regions that affect pain, cognition, motivation, and stress responses, including the anterior cingulate cortex (ACC), prefrontal cortex, hippocampus, and nucleus accumbens. Therefore, it plays a central role in emotional and motivational processing [16–18]. An imbalance between excitation and inhibition (E/I) in the BLA, such as hyperexcitability, induces anxiety [19, 20]. Inflammation is another factor that can affect the onset and development of anxiety in the amygdala [21, 22]. This study investigates the effects of FMNT on the treatment of chronic pain and anxiety. Open field (OF) and elevated plus maze (EPM) anxiety tests, as well as the Von Frey and hot plate pain tests were conducted on a model group of mice injected with complete Freund’s adjuvant (CFA) to simulate pain- and anxiety-like behaviors. The influence of FMNT in the BLA, and the underlying mechanisms, were closely examined.

## Materials and methods

### Animals

C57BL/6 male mice (age 7–8 weeks) from the Laboratory Animal Center of the Fourth Military Medical University (FMMU) were used in all of the experiments. The mice were divided into four groups, and each group was kept in a separate cage under standard laboratory conditions (12 h light/12 h dark, temperature 22–26 °C, humidity 55–60%) with water and food provided ad libitum. Mice in the control group were not chemically treated in any way. The CFA group of mice was given single-dose injections of CFA, while the CFA + FMNT group was injected first with CFA, then with FMNT. FMNT group was only treated with FMNT. Prior to the beginning of the experiment, the animals were permitted to acclimate to the laboratory environment for at least 1 week. All of the experiments were carried out in accordance with protocols approved by the Institutional Ethical Committee of the FMMU.

### Chronic inflammatory pain mouse model and drug treatment

To induce chronic inflammatory pain, a single dose of CFA (50% CFA, 10 μl; Sigma, St. Louis, MO, USA) was injected into the plantar surface of the right hind paws [23] of mice in the CFA and CFA + FMNT groups. One week after CFA injection, CFA + FMNT mice were administered with FMNT (purity ≥98%, Aladdin, Shanghai, China) by intraperitoneal injection (i.p) at a dose of 25 mg/kg, once a day for 8–10 consecutive days. FMNT was dissolved in olive oil to a concentration of 10 mg/ml. Equal volume olive oil was intraperitoneally injected into the control and CFA mice. On the day of behavior test and sample preparation, FMNT, or olive oil, was administrated 30 min before commencing with the testing procedure.

### Open field test

The open field (OF) test was carried out to detect anxiety-like behavior in CFA-injected mice as described in previous work [24]. The OF, a square arena (30 cm × 30 cm × 30 cm) with plastic walls and floor, was placed inside an isolated chamber with illumination. Mice were put into the central area of the box and allowed to freely explore for 15 min. Movement loci of mice were recorded using a camera fixed above the floor, and analyzed with a video-tracking system (DigBehv-LR4, Shanghai Jiliang, China). The OF test was performed before the elevated plus maze (EPM) test on the same day.

### Elevated plus maze (EPM)

To further evaluate anxiety-like behavior, EPM tests were also conducted, as detailed in a previous study [19]. Briefly, the apparatus (RD1208-EP, Shanghai Mobiledatum Corporation, China) consists of two open arms (25 cm × 8 cm × 0.5 cm) and two closed arms (25 cm × 8 cm × 12 cm) that extend from a common central platform (8 cm × 8 cm), placed at a height of 50 cm above the floor. Mice were allowed to habituate to the testing room for 24 h before the test. For each test, an individual mouse was placed in the center square, facing an open arm, and allowed to explore freely for 5 min. The degree of anxiousness was evaluated based on the number of entries into and the time spent in the open arms [25]. An entry was defined as having all four paws placed inside an arm. Mice movement was monitored using a video-tracking system composed of a camera fixed above the maze.

### Von Frey test

This test was conducted to assess the pain threshold in mice. The setup consists of a plastic box with a metal mesh floor. The mice were individually placed inside this box and allowed to adjust to the environment for 30 min before testing. Using Dixon’s up-down paradigm, the sensitivity of mechanical allodynia was determined based on the responsiveness of the hind paw to the point of bending of von Frey filaments. In this study, filaments with different bending forces (0.008–2 g) were applied to the middle of the hind paw dorsum in an ascending order. Licking, biting, and sharp withdrawal of the hind paw were considered as positive responses. A rest interval of at least 3 minutes was allowed between consecutive stimulations. The results were tabulated and the pain threshold was assigned at 50% withdrawal.

### Hot plate test

To assess thermal nociceptive responses, a commercially available plantar analgesia instrument (BME410A, Institute of Biological Medicine, Academy of Medical Science, China) was employed. Again, the mice were individually placed in plastic boxes and allowed to acclimate for 30 min. Thermal hyperalgesia was assessed by measuring the latency of paw withdrawal (PWL), defined as the time extending from radiant heat application to withdrawal of the hind paw [26]. The heat source was turned off automatically when the mouse lifted its foot. In order to prevent tissue damage, the heat source was automatically cut off at 40 s even if the mouse did not lift its hind paw. The experiment was repeated five times, with a five-minute rest interval between two consecutive tests.

### Western blot analysis

**Fig. 1:**
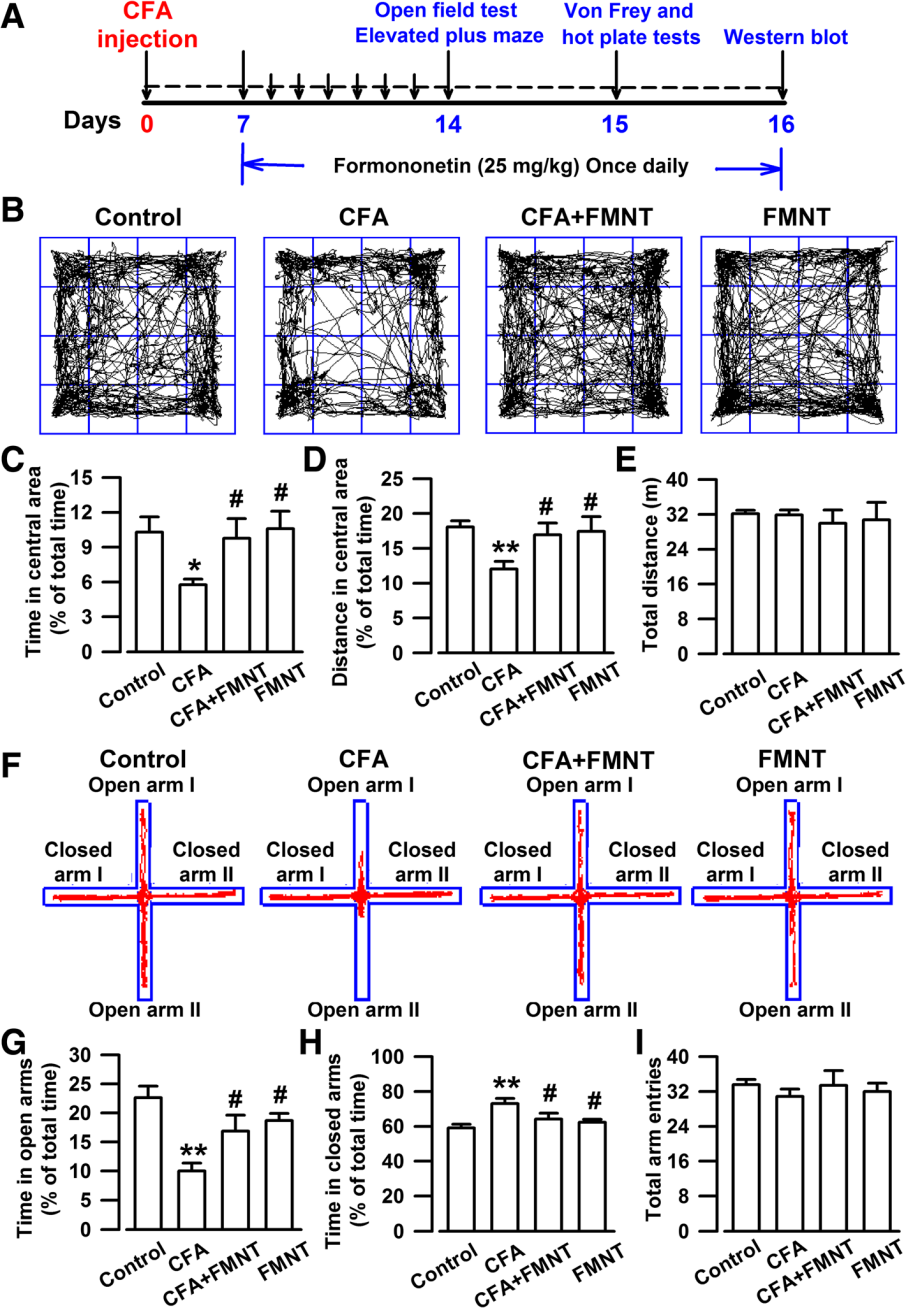
FMNT relieved anxiety-like behaviors in mice injected with CFA. **a** Schedule showing the experimental procedure. **b** Representative traces in OF test during a period of 15 min. Behavioral tests were performed on Day 14. **c-e** In OF test, administration with FMNT (25 mg/kg) for 8 days significantly increased the time (**c**) and distance (**d**) spent in the central area but had no effects on the total traveled distance (**e**). **f** Representative traces in EPM test during a period of 5 min. **g-i** FMNT treatment reversed the time spent in open arms (**g**) and closed arms (**h**). However, total arm entries had no difference among four groups (**i**). *n* = 7 per group. ^*^p < 0.05, ^**^p < 0.01 vs. control; ^**#**^*p* < 0.05 vs. CFA

On the 16th day after CFA injection (Fig. 1a), at 30 min after the administration of FMNT in CFA + FMNT and FMNT groups, all mice were anesthetized with 4% isoflurane and then decapitated. Coronary slices (300 μm) of their extracted brains were obtained by Vibratome, and the bilateral BLA were isolated under anatomical microscope. Western blot analysis was performed as detailed in Liu et al. [27]. The BLA sample was dissociated via sonication in RIPA lysis buffer containing phosphatase and protease inhibitors. The protein content of the collected samples was quantified using the BCA Protein Assay Kit. Equal amounts of protein (40 μg) were dispersed on SDS-PAGE gels then electro-transferred to PVDF membranes (Invitrogen). The latter were in turn probed with antibodies after incubation for 1.5 h in 5% non-fat milk. The antibodies used are Anti-β-actin (1:50000; A5316) purchased from Sigma (St. Louis, MO, USA); Anti-Iba-1 (1: 1:1000; ab178847), anti-GluN2B (1:1000; ab65783), anti-phosphorylated GluN2B at the S1303 site (p-GluN2B-S1303; 1:1000; ab81271), anti-GluA1 (1:1000; ab31232), anti-PSD95 (1:1000; ab2723), and anti-GABA_A_α2 (1:1000; ab72445) from Abcam (Cambridge, UK); Anti-GluN2A (1:1000; ab1555), anti-phosphorylated GluA1 at the S845 site (p-GluA1-S845; 1:1000; ab5849), and anti-phosphorylated GluA1 at the S831 site (p-GluA1-S831; 1:1000; ab5847) from Millipore (Billerica, MA, USA); Anti-NF-κB p65 (1:750; AF0874) from Affinity Biosciences (USA); Anti-GABA_A_γ2 (1:500; BS6858) from Bioworld (St. Louis Park, MN, USA). The following antibodies were purchased from Cell Signaling Technology (Danvers, MA, USA): glial fibrillary acidic protein (GFAP; 1:1000; #3670), anti-phosphorylated GluN2B at the T1472 site (p-GluN2B-T1472; 1:1000; #4208 s), anti-cAMP-response element binding protein (CREB; 1:1000; #9197), and anti-phosphorylated CREB (p-CREB; 1:1000; #9198). The membranes were further incubated in media containing horseradish peroxidase-conjugated secondary antibodies (anti-rabbit/anti-mouse IgG for the primary antibodies, Santa Cruz, CA, USA). All of the chemicals and reagents were commercially available with standard biochemical quality. Densitometric analysis of Western-blot was conducted using a ChemiDoc XRS (Bio-Rad, Hercules, CA) and quantified using Image J software (NIH, Bethesda, Maryland), according to the instructions. For data analysis, the band intensity of each blot was calculated as a ratio, using β-actin as reference. The intensity ratio for the control group was set at 100%, and the intensity ratios of other treatment groups were expressed as relative percentages.

### In silico docking study of Formononetin with NMDA receptor and CREB binding protein

A study of in silico docking of FMNT with NMDA receptor and CREB binding protein was conducted. For this purpose, different ligands were prepared and optimized using the Prepare Ligands module, a protocol of Discovery Studio 3.5 (Accelrys Inc.), then converted to the SD file format. NMDA receptor (NMDAR, PDB code: 4PE5) and CREB binding protein (CBP, PDB code: 5CGP) structures were downloaded from the RCSB Web site (http://www.pdb.org) in PDB format. Before docking, the original crystal ligands and water molecules were removed from the protein-ligand complexes. Hydrogen atoms were added by application of CHARMM force field [28] and the Momany-Rone partial charge [29] default settings in Discovery Studio 3.5. The ligand-binding site was extracted from the PDB site. Docking analyses of Formononetin with the NMDAR or CBP in the presence of crystal ligands were performed by means of the CDOCKER module, which is accurate when active sites are known. This method meets the requirements of experimental verification. The number of generated poses was set to 100 for each ligand, and default settings were selected for other parameters.

### Statistical analysis

The obtained data values are presented as mean ± SEM. Statistical analysis of multiple groups was performed by one-way analysis of variance (ANOVA) followed by least significant difference (LSD) test or Dunnett’s test for post hoc comparisons (SPSS 13.0). In all cases, *p* < 0.05 was considered to be statistically significant.

## Results

### Effect of FMNT on anxiety-like behavior

The anxiety-like behavior in mice was assessed using OF and EPM tests performed on the 14th day (Fig. 1a). In the case of OF testing, it was found that mice in the control group moved longer distances (F_3,24_ = 3.976, *P* = 0.005, Fig. 1b and d), and for longer periods (F_3,24_ = 3.026, *P* = 0.018, Fig. 1b and c), in the central area of the setup, than CFA-injected mice. Moreover, mice in the control group spent more time in the open arms (F_3,24_ = 6.918, *P* < 0.001, Fig. 1f and g), and less time in the closed arms (F_3,24_ = 4.826, *P* = 0.001, Fig. 1f and h) of the EPM test setup than the CFA-injected mice. The administration of FMNT at a dose of 25 mg/kg for 8 consecutive days markedly increased the time (F_3,24_ = 3.026, *P* = 0.034, Fig. 1c) and distance (F_3,24_ = 3.976, *P* = 0.019, Fig. 1d) traveled in the central area, as well as the time spent in the open arms (F_3,24_ = 6.918, *P* = 0.024, Fig. 1g), while decreasing the time spent in the closed arms (F_3,24_ = 4.826, *P* = 0.03, Fig. 1h). The effects of FMNT in OF and EPM tests were found to be dose-dependent (Additional file 1: Figure S1). The total distance traveled in OF tests and the total arm entries in EPM tests were comparable among the investigated groups of mice, indicating that normal locomotor activity is maintained after CFA injection and FMNT treatment (F_3,24_ = 0.197, *P* = 0.897, Fig. 1e; F_3,24_ = 0.338, *P* = 0.798, Fig. 1i; F_4,25_ = 0.332, *P* = 0.854, Additional file 1: Figure S1c; F_4,25_ = 0.022, *P* = 0.999, Additional file 1: Figure S1f). These results suggest that FMNT treatment has anxiolytic effects in mice injected with CFA.

### Effect of FMNT on pain-like behavior

**Fig. 2:**
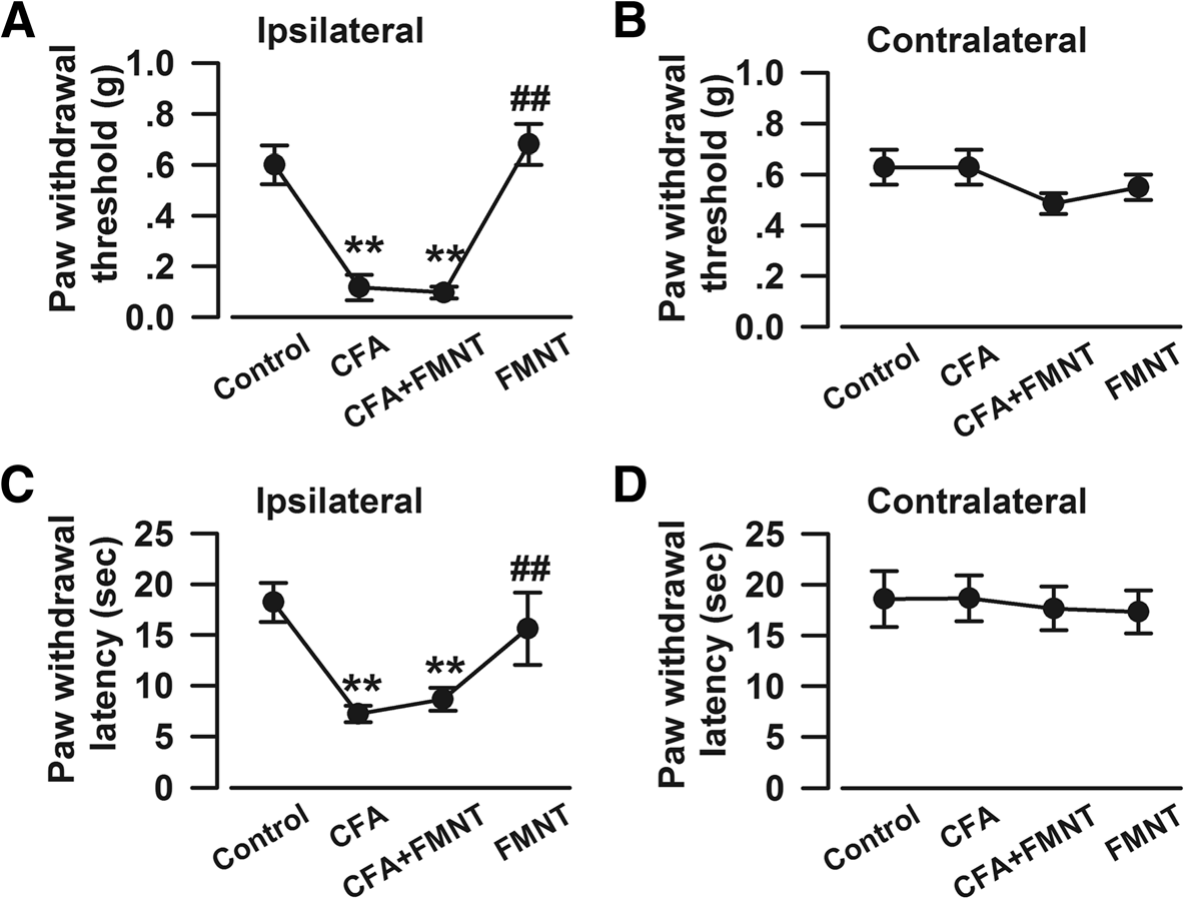
FMNT had no analgesic effects in mice with chronic inflammatory pain. Von Frey and hot plate tests were performed on Day 15 (Fig. 1a). FMNT (25 mg/kg) did not alleviate CFA-induced mechanical allodynia (**a**) and thermal hyperalgesia (**c**) in CFA-injected hind paw (ipsilateral). The basal mechanical allodynia (**b**) and thermal hyperalgesia (**d**) in contralateral hind paw were not affected by CFA and/or FMNT. *n* = 7 per group. ^**^p < 0.01 vs. control; ^**##**^*p* < 0.01 vs. CFA

Von Frey and hot plate tests were performed on the 15th day after CFA injection (Fig. 1a) to assess mechanical allodynia and thermal hyperalgesia effects, respectively. The threshold (F_3,24_ = 27.671, *P* < 0.001, Fig. 2a) and latency (F_3,24_ = 10.037, *P* < 0.001, Fig. 2c) of ipsilateral paw withdrawal were significantly reduced in CFA-injected mice, as compared to the control group. Surprisingly, FMNT treatment had no significant effect on either criterion (F_3,24_ = 27.671, *P* = 0.82, Fig. 2a; F_3,24_ = 10.037, *P* = 0.537, Fig. 2c), even at increased dosage (Additional file 1: Figure S1a and c). Moreover, neither CFA nor FMNT affected the threshold (F_3,24_ = 1.415, *P* = 0.266, Fig. 2b; F_4,25_ = 0.694, *P* = 0.603, Additional file 1: Figure S1b) and latency (F_3,24_ = 0.074, *P* = 0.973, Fig. 2d; F_4,25_ = 0.598, *P* = 0.667, Additional file 1: Figure S1d) of the contralateral paw withdrawal. These results confirm that CFA induces mechanical allodynia and thermal hyperalgesia; however, FMNT is shown to have no analgesic effect in mice.

### Effect of FMNT on CFA-induced upregulations of NMDA receptors and CREB in the BLA

**Fig. 3:**
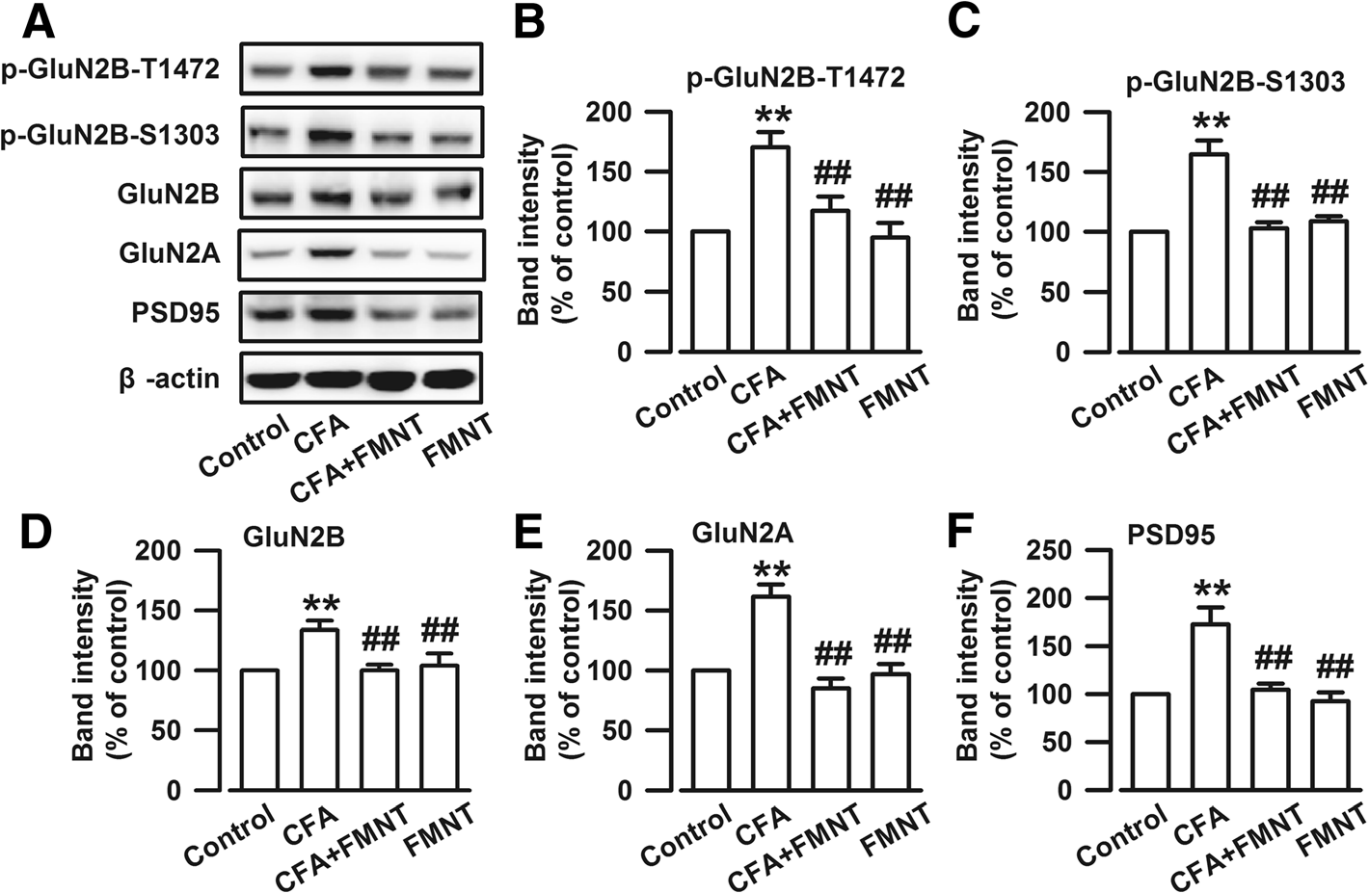
FMNT reduced the CFA-induced upregulations of NMDA receptors in the BLA. **a** Representative Western blot analysis of p-GluN2B-T1472, p-GluN2BS1303, GluN2B, GluN2A, and PSD95. FMNT (25 mg/kg) treatment for 10 days reversed the up-regulations of p-GluN2B-T1472 (**b**), p-GluN2BS1303 (**c**), GluN2B (**d**), GluN2A (**e**), and PSD95 (**f**). *n* = 6 per group. **p < 0.01 vs. control; ^##^*p* < 0.01 vs. CFA

The following experiments were focused on the BLA of amygdala, because, according to behavioral studies, the BLA is implicated in the onset and development of anxiety [30], a behavioral abnormality that has been associated with synaptic changes [31] and expression alteration of NMDAR [32]. The levels of NMDAR in the BLA of mice were examined on the 16th day (Fig. 1a). CFA injection evidently increased phosphorylated GluN2B at T1472 (p-GluN2B-T1472, F_3,20_ = 10.352, *P* < 0.001, Fig. 3a and b) and S1303 (p-GluN2B-S1303, F_3,20_ = 20.309, *P* < 0.001, Fig. 3a and c), GluN2B (F_3,20_ = 5.79, *P* = 0.002, Fig. 3a and d), GluN2A (F_3,20_ = 19.168, *P* < 0.001, Fig. 3a and e), and PSD95 (F_3,20_ = 12.359, *P* < 0.001, Fig. 3a and f) levels. The upregulations of p-GluN2B-T1472 (F_3,20_ = 10.352, *P* = 0.002, Fig. 3a and b), p-GluN2B-S1303 (F_3,20_ = 20.309, *P* < 0.001, Fig. 3a and c), GluN2B (F_3,20_ = 5.79, *P* = 0.002, Fig. 3a and d), GluN2A (F_3,20_ = 19.168, *P* < 0.001, Fig. 3a and e), and PSD95 (F_3,20_ = 12.359, *P* < 0.001, Fig. 3a and f) were reversed after FMNT treatment. FMNT administration alone had no effect on the levels of these proteins (Fig. 3a-f).

**Fig. 4:**
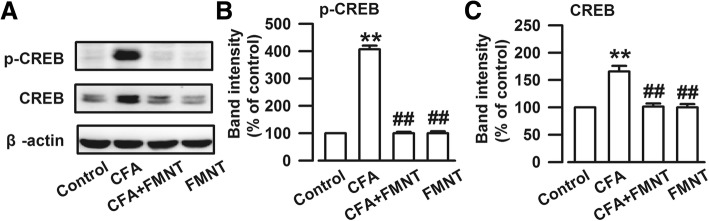
FMNT inhibited CREB signaling pathway in the BLA of mice with CFA injection. **a** Representative Western blot analysis of p-CREB and CREB. The expressions of p-CREB (**b**) and CREB (**c**) were significantly increased in the BLA of mice with CFA injection, which were reversed after FMNT treatment. *n* = 6 per group. **p < 0.01 vs. control; ^##^*p* < 0.01 vs. CFA

The cAMP-response element binding protein (CREB) is activated by the NMDA receptor [33]. The expressions of phosphorylated CREB (p-CREB, F_3,20_ = 356.521, *P* < 0.001, Fig. 4a and b) and total CREB (F_3,20_ = 24.019, *P* < 0.001, Fig. 4a and c) increased significantly upon CFA injection. These elevated protein levels were abolished by FMNT treatment (F_3,20_ = 356.521, *P* < 0.001, Fig. 4b; F_3,20_ = 24.019, *P* < 0.001, Fig. 4c). The obtained results suggest that FMNT treatment might relieve anxiety-like behavior by inhibiting NMDA/CREB signaling pathways.

### Structural interactions of FMNT with NMDAR and CBP

**Fig. 5:**
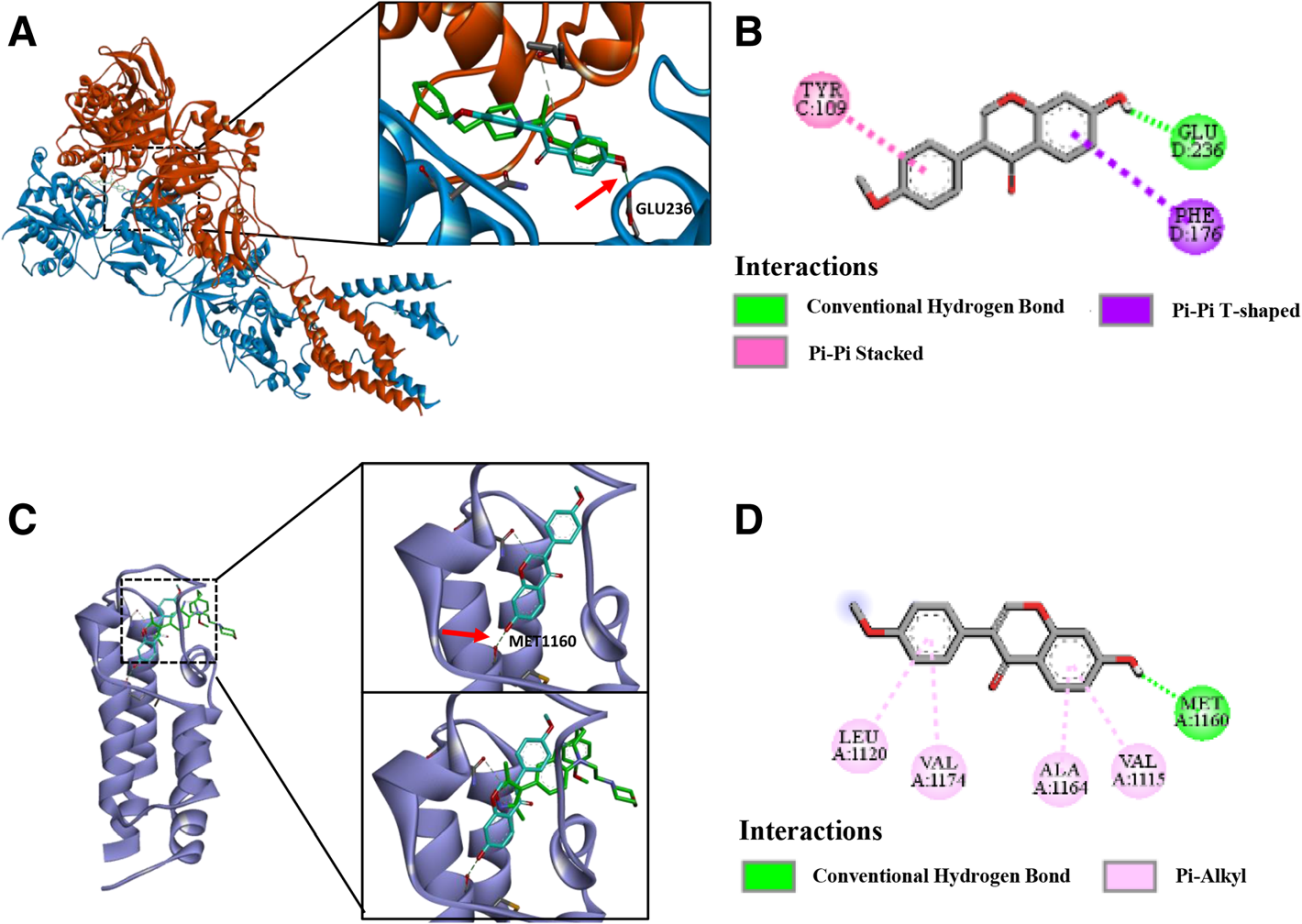
Structural interactions of FMNT with NMDA receptor and CBP. **a** Structural representation of FMNT binding to NMDA receptor (PDB:4PE5; GluN2B: blue ribbon; GluN1a: red ribbon) as inferred from docking simulations (left). Ifenprodil was shown as green sticks, and FMNT was shown as cyan sticks (right). The -OH group of FMNT formed hydrogen-bond with Glu236 of NMDA receptor (red arrow in right). The best-docked pose of FMNT in the active site coincided well with the crystal ligand ifenprodil (NMDA antagonist). **b** 2D diagram of interaction between FMNT and NMDA receptor showed the major binding sites and bonding forces. **c** Characterization of spatial interactions within the FMNT-CBP (PDB:5CGP, CBP: bluish violet ribbon) complex (left). FMNT was shown as cyan sticks (top-right) and crystal ligand BDOIA383 (ligand of CBP) was shown as green sticks (bottom-right). The -OH group of the FMNT formed hydrogen-bond with Met1160 of CBP (red arrow in the top-right). **d** 2D diagram showed the major binding sites and bonding forces between FMNT and CBP

The results reported in the preceding section suggest that the NMDA receptor may be an important target for FMNT. To further investigate the potential mechanism by which FMNT regulates anxiety-like behavior, molecular docking analysis of this compound was conducted. FMNT (cyan sticks in Fig. 5a) was docked to the NMDA receptor (red and blue ribbon in Fig. 5a) by means of the CDOCKER module of Discovery Studio (Accelrys Inc., San Diego, CA, USA). The OH group at the chromen-one site of FMNT (red arrow in the right of Fig. 5a) interacts with NMDAR at the GLU236 site (green symbol in Fig. 5b) via hydrogen-bonding, which coincides well with the crystal ligand ifenprodil, an NMDA receptor antagonist (green sticks in Fig. 5a). This suggests that FMNT could bind to GluN2B. Moreover, the phenyl moiety of FMNT can form π-π stacked interactions with TYR109, thus, contributing to the stability of the protein-ligand complex (pink symbol in Fig. 5b). The superposition between the crystal structures of CBP-BDOIA383 (green sticks, BDOIA383: ligand of CBP) and FMNT (cyan sticks) is shown in Fig. 5c. The two compounds exhibit totally different conformations and interactions with CBP (bluish violet ribbon in Fig. 5c). In particular, the OH group at the chromen-one moiety of FMNT (red arrow in the top-right of Fig. 7c) is deeply projected towards MET1160 of CBP (green symbol in Fig. 5d), and forms a hydrogen-bonding interaction with it. This is not observed in the case of crystal ligand BDOIA383 (bottom-right in Fig. 5c). These results indicate that FMNT binds more strongly to CBP than to BDOIA383.

### Effect of FMNT on the CFA-induced upregulations of AMPA receptor in the BLA

**Fig. 6:**
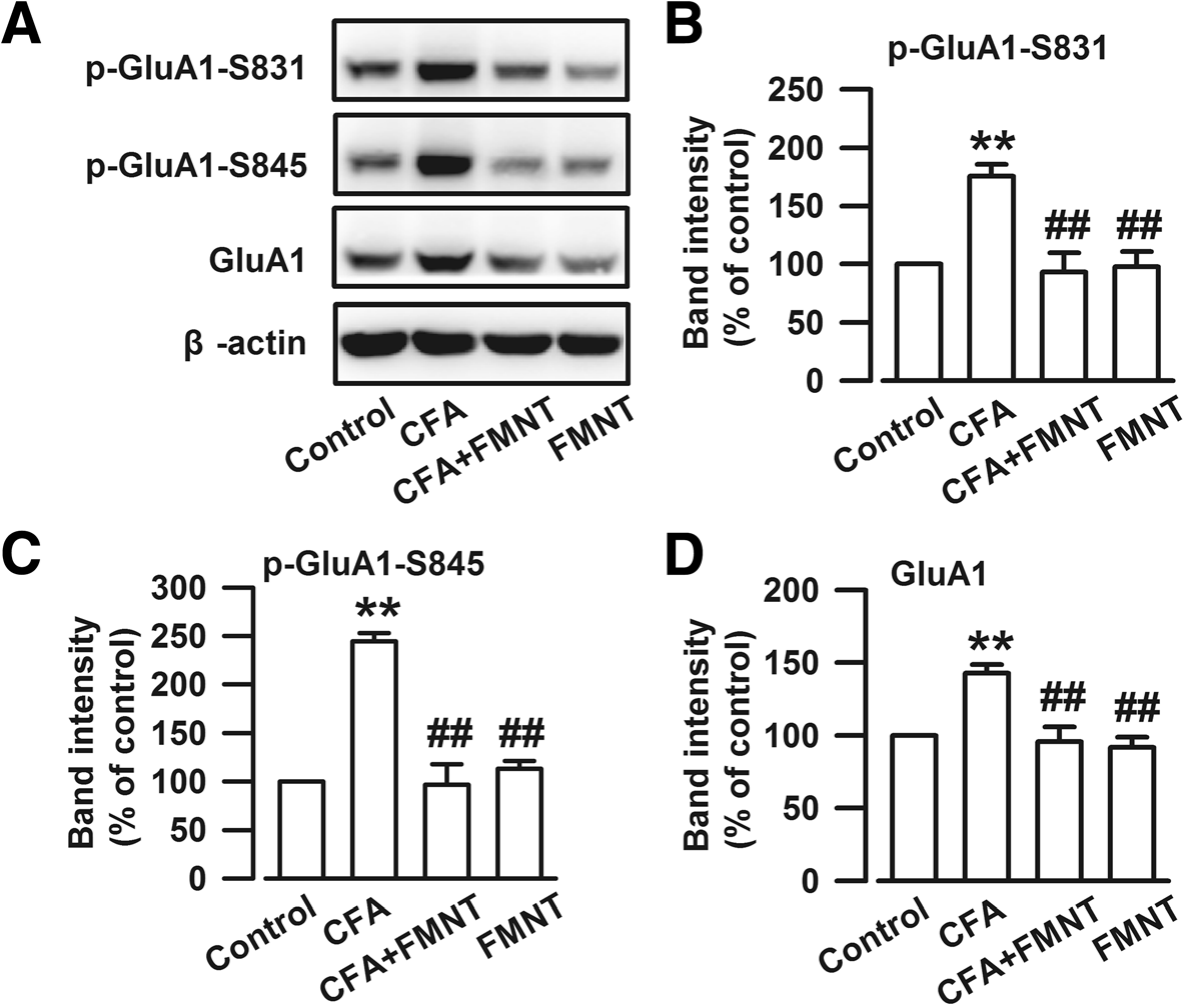
FMNT reversed the increased level of AMPA receptor in the BLA induced by CFA. **a** Representative Western blot analysis of p-GluA1-S831, p-GluA1-S845, and GluA1. CFA injection increased the expressions of p-GluA1-S831 (**b**), p-GluA1-S845 (**c**), and GluA1 (**d**), and FMNT (25 mg/kg) markedly reduced the overexpression of -GluA1-S831 (**b**), p-GluA1-S845 (**c**), and GluA1 (**d**) in the BLA of CFA-injected mice. *n* = 6 per group. **p < 0.01 vs. control; ^##^*p* < 0.01 vs. CFA

The activation of CREB leads to the phosphorylation of GluA1-containing AMPA [34], another important glutamate receptor closely related to the regulation of anxiety [35]. Therefore, the expression of the AMPA receptor subunit GluA1 was examined, and it was found that the levels of phosphorylated GluA1 at S831 (p-GluA1-S831, F_3,20_ = 11.363, *P* < 0.001, Fig. 6a and b) and S845 (p-GluA1-S845, F_3,20_ = 35.255, *P* < 0.001, Fig. 6a and c), as well as total GluA1 (F_3,20_ = 11.906, *P* < 0.001, Fig. 6a and d), were significantly enhanced in the BLA of mice after CFA injection. FMNT administration resulted in the downregulation of p-GluA1-S831 (F_3,20_ = 11.363, *P* < 0.001, Fig. 6a and b), p-GluA1-S845 (F_3,20_ = 35.255, *P* < 0.001, Fig. 6a and c), and GluA1 (F_3,20_ = 11.906, *P* < 0.001, Fig. 6a and d) expressions, but had no influence on the levels of phosphorylated and total GluA1. These results implied that the antianxiety effect of FMNT was related to the inhibition of AMPA receptors in the BLA.

### Effect of FMNT on CFA-induced upregulations of GABA_A_ receptors in the BLA

**Fig. 7:**
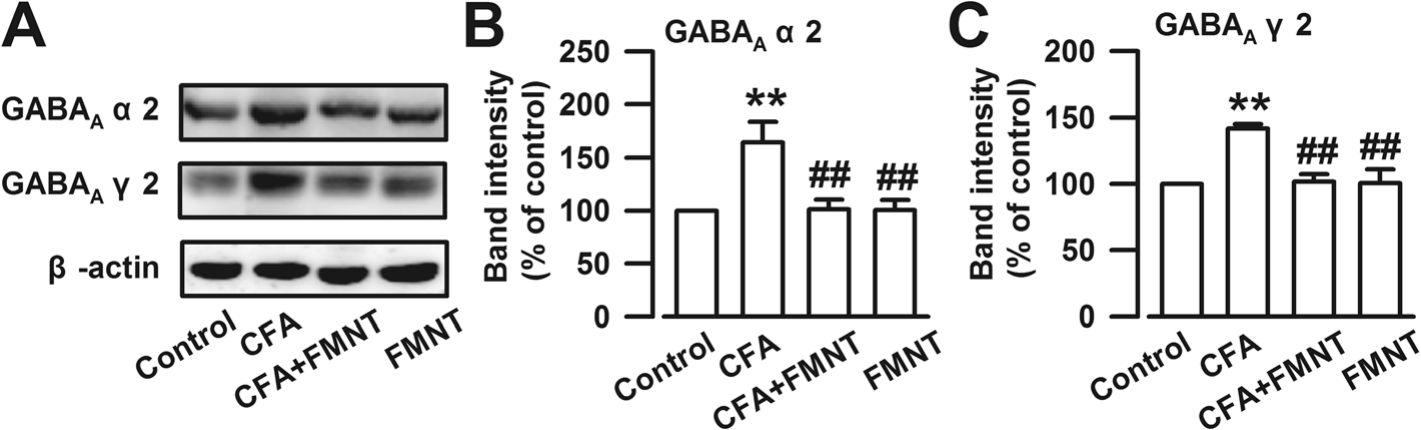
FMNT blocked CFA-induced upregulations of GABA_A_ receptors in the BLA. **a** Representative Western blot for GABA_A_ α2 and GABA_A_ γ2. The levels of GABA_A_ α2 (**b**) and GABA_A_ γ2 (**c**) were significantly increased in the BLA of CFA-injected mice, which were downregulated by FMNT (25 mg/kg). n = 6 per group. **p < 0.01 vs. control; ^##^p < 0.01 vs. CFA

The GABA_A_ receptor is a crucial drug target for anxiolytics such as benzodiazepines. Therefore, the expressions of GABA_A_ α2 and GABA_A_ γ2, two subunits of the GABA_A_ receptor in the BLA, were assessed. The obtained results show that the levels of GABA_A_ α2 (F_3,20_ = 7.493, *P* = 0.001, Fig. 7a and b) and GABA_A_ γ2 (F_3,20_ = 11.281, *P* < 0.001, Fig. 7a and c) were unexpectedly enhanced in the BLA of mice after CFA injection. These increased expressions were blocked upon treatment with FMNT. It may be concluded that alteration of the GABA_A_ receptor is part of the regulating effect of FMNT on anxiety in the BLA.

### Effect of FMNT on the NF-κB signaling pathway and microglia activation in the BLA

**Fig. 8:**
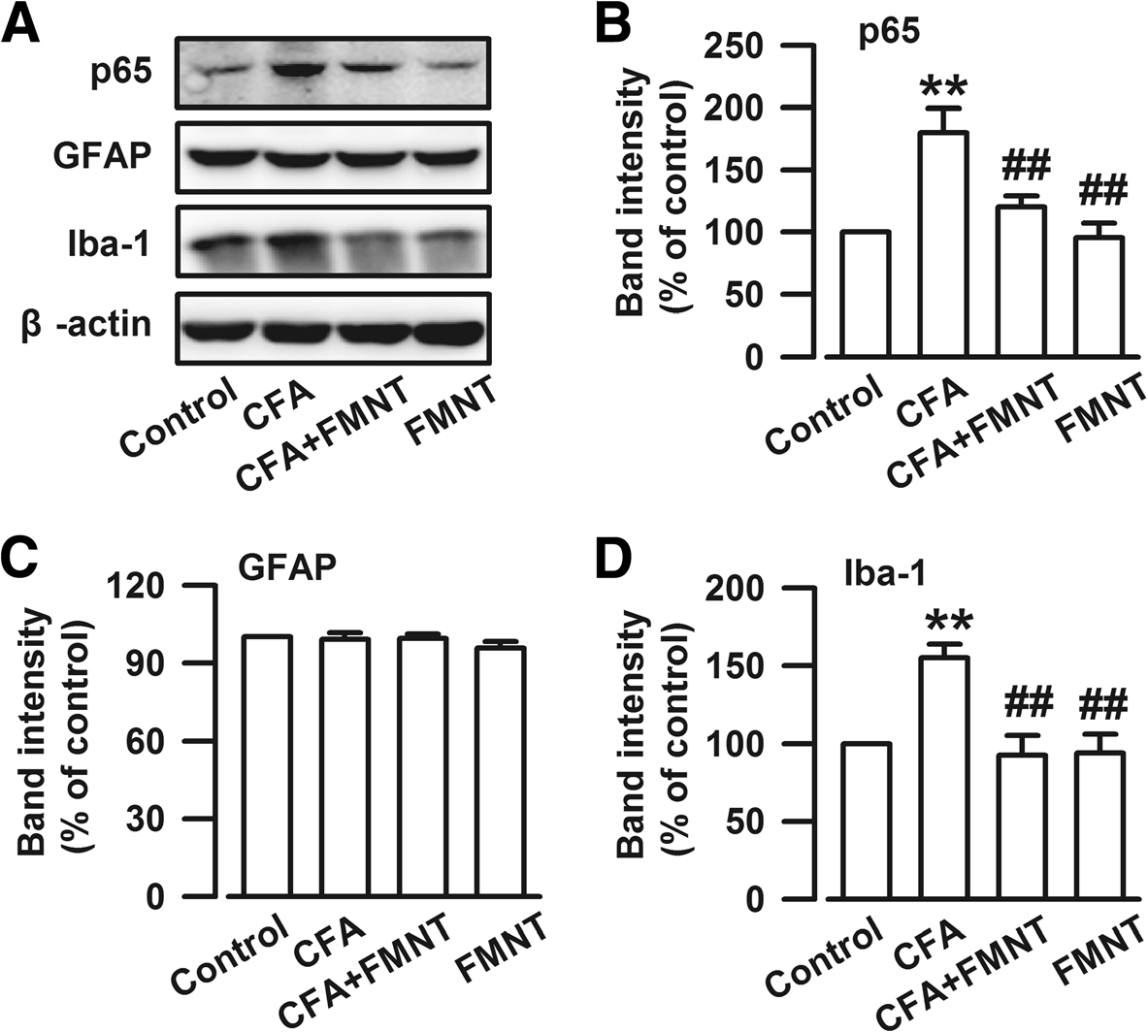
FMNT inhibited NF-κB signaling pathway and microglia activation in the BLA. **a** Representative bands of Western blot analysis showing the levels of NF-κB p65, GFAP, and Iba-1. CFA injection significantly increased NF-κB p65 (**b**) and Iba-1 (**d**) levels in the BLA. However, GFAP levels were not changed among four groups (**c**). FMNT (25 mg/kg) notably reversed the overexpression of NF-κB p65 (**b**) and Iba-1 (**d**). *n* = 6 per group. **p < 0.01 vs. control; ^##^*p* < 0.01 vs. CFA

Inflammation plays a key role in anxiety [36]. Thus, it is important to assess whether or not FMNT treatment is related to inflammatory inhibition as part of its anxiolytic effect. For this purpose, the levels of NF-κB p65, a protein complex in the BLA that plays an important role in regulating the immune response to infection [37], were determined. The results showed that CFA injection (F_3,20_ = 10.434, *P* < 0.001, Fig. 8a and b) upregulated these levels, and that this effect was reversed by FMNT treatment (F_3,20_ = 10.434, *P* = 0.002, Fig. 8a and b). Furthermore, knowing that the activation of astrocyte and microglia is required for the onset and progression of inflammation in the central nervous system (CNS) [38, 39], these cells were also examined. Specifically, GFAP and Iba-1 markers of astrocyte and microglia, respectively, which are known to be upregulated during inflammation period [40], were monitored. Interestingly, Iba-1 levels (F_3,20_ = 9.427, *P* = 0.001, Fig. 8a and d) were enhanced in the BLA of CFA-injected mice, but not GFAP levels (F_3,20_ = 0.955, *P* = 0.717, Fig. 8a and c). FMNT administration reduced the expression of Iba-1 (F_3,20_ = 9.427, *P* < 0.001, Fig. 8a and d), but had no effect on GFAP levels (F_3,20_ = 0.955, *P* = 0.867, Fig. 8a and c). These results suggest that FMNT alleviates inflammation-induced anxiety-like behaviors by blocking the NF-κB signaling pathway and microglia activation.

## Discussion

Many studies have shown that chronic pain is often accompanied with anxiety. For example, lipopolysaccharide (LPS)-induced pulmonary inflammation is accompanied with anxiety-like behavior in rats [41]. Chronic pain acts as an inescapable stressor on the hypothalamo-pituitary-adrenal axis to induce emotional disorder [42, 43]. Therefore, pain and emotional disorder possibly share the same biological pathways and neurotransmitters, which influences concurrent treatments [44, 45]. In the present study, CFA-injected mice exhibited obvious pain and anxiety-like behaviors. Treatment with FMNT, a medicinal drug extracted from *Trifolium pratense* L., relieved CFA-induced anxiety-like behaviors in mice, but had no effect in alleviating pain-related behaviors. Knowing that the amygdala is an important brain area for anxiety modulation [46, 47], and that the BLA of the amygdala is involved in the development of anxiety in mice [19], our research regarding the effect of FMNT on pain and anxiety was performed in the BLA.

The excitatory/inhibitory (E/I) neuronal network maintains a finely tuned balance of neural activity that is critical for central physiological function. Imbalance of E/I signaling instigates patterns of seizure, schizophrenia, and autism [48, 49]. NMDA and AMPA are crucial excitatory postsynaptic receptors that exhibit enhanced activity due to neurotransmitter hyperexcitability, a condition that is associated with increased anxiety [50]. PSD95 is a postsynaptic anchor protein that binds to NMDA and AMPA receptors [51]. In this study, it was shown that CFA injection results in the upregulation of NMDA receptors, AMPA receptors, and PSD95, which leads to increased excitability in the BLA, thereby instigating anxiety-like behavior in mice. Moreover, excitatory activity in the BLA is tightly regulated by a relatively small population of GABAergic inhibitory neurons [52]. Among the three subtypes of GABA receptors (GABA_A_, GABA_B,_ and GABA_C_ subtype), GABA_A_ receptors are typical ligand-gated ion channels that play the most important role in GABAergic inhibitory function, which is closely connected with anxiety modulation [17, 50]. Reduced GABAergic inhibition in the BLA usually underlies anxiety disorders. For example, postpartum estrogen withdrawal impairs GABA_A_ receptor-mediated inhibition in the BLA and causes anxiety [53]. Two highly expressed subunits of GABA_A_ receptors—GABA_A_α2 and GABA_A_γ2—were examined in this project. The levels of these receptors were surprisingly enhanced in the BLA of CFA-treated mice, then returned to normal after FMNT treatment. The results obtained in this study are consistent with those reported previously [24] concerning the protective effect of GABA_A_α2 and GABA_A_γ2 upregulations on the E/I balance in the BLA of CFA-injected mice. FMNT reduces the neural excitability and the protective upregulation of GABA_A_ receptors.

In silico docking analyses conducted using computer-assisted drug design showed that FMNT could bind to NMDA receptors (NMDAR) as well as CREB binding proteins (CBP). In this work, the binding potential of FMNT was explored only at the active site, since docking to this site makes it easy to speculate whether the compound is active or not. Interestingly, FMNT had better potential interactions with CBP than BDOIA383, a reported inhibitor of CBP [54]. This suggests that FMNT may inhibit CREB-mediated gene transcription by binding to CBP. Studies have demonstrated that CREB-dependent transcription is essential for both long-lasting forms of synaptic plasticity and long-term memory [55–57]. CREB activation can also directly control neuronal excitability [58]. However, we are unable to guarantee that FMNT does not have an interaction with other non-explored binding sites of these two proteins (NMDAR and CBP). The development of methods and programs, such as BINDSURF [59], METADOCK [60], LeadFinder [61, 62], BLIND DOCKING SERVER, Autodock Vina [63], and FlexScreen [64, 65], allows for more comprehensive and accurate docking analyses. In future research, we will use full blind docking methods to explore the real binding mode and possibility. Meanwhile, owing to multi-target effects of traditional Chinese medicine, other drug targets of FMNT cannot be ruled out.

Knowing that inflammation plays a key role in the development of anxiety [66], the anti-inflammatory effects of FMNT were also explored by monitoring the expressions of (i) NF-κB, the first responder to inflammation, (ii) microglia, dynamic immune cells of the brain that elicit an immune response during brain damage, and (iii) astrocytes, neural cells that produce pro-inflammatory cytokines and enhance neuronal damage [38]. In the present study, it was found that CFA injection markedly increased NF-κB p65 levels and activated microglia in the BLA. These effects were inhibited upon FMNT treatment. The expression of GFAP did not change after CFA injection and/or FMNT treatment, indicating that the astrocytes were not involved in the modulation of anxiety, and that the anxiolytic effects of FMNT may be related to the inhibition of microglia activation by NF-κB p65 signaling pathway. Interestingly, FMNT did not affect pain-like behavior although it had an anti-inflammatory effect in the BLA. This suggests that the anti-inflammatory effect of FMNT is strong enough for emotional regulation, but not for pain modulation.

CFA-injected mice exhibited obvious pain- and anxiety-like behavior. However, this anxiety model is different from stress-induced, drug-induced, or social anxiety. Therefore, although we were able to prove that FMNT has the potential to diminish pain-induced anxiety, its effect on the other models still needs to be assessed. CFA-generated inflammatory pain may stimulate the activation of many brain regions, such as the BLA, ACC, hippocampus, and nucleus accumbens [67–70]. So the effects of FMNT on other brain regions may also contribute to its anxiolytic effect. Furthermore, we cannot exclude other mechanisms and signaling pathways that may also be involved in the anxiolytic effect of FMNT.

In summary, the data collected in this work provide strong evidence for the anxiolytic effect of FMNT in mice suffering from chronic inflammatory pain. The fundamental mechanisms of this effect rely on the inhibition of hyperexcitability and inflammation in the BLA.

## Additional file

Additional file 1: **Figure S1.** Anxiolytic effect of FMNT was dose-dependent in mice injected with CFA. **Figure S2.** Different dosages of FMNT had no analgesic effects in mice injected with CFA. (PDF 243 kb)

## Abbreviations

BLA: Basolateral amygdala
CBP: CREB binding protein
CFA: Complete Freund’s adjuvant
CREB: cAMP-response element binding protein
E/I: Excitatory/inhibitory
EPM: Elevated plus maze
FMNT: Formononetin
GFAP: Glial fibrillary acidic protein
NMDA: N-methyl-D-asparate
NMDAR: NMDA receptor
OF: Open field
