# Supplementary material for: Anxiolytic effects of Formononetin in an inflammatory pain mouse model
Source: Mol Brain. 2019 Apr 8;12:36. doi: 10.1186/s13041-019-0453-4 (PMC6454770; doi:10.1186/s13041-019-0453-4)
Supplement: Supplementary file 1 — Figure S1. Anxiolytic effect of FMNT was dose-dependent in mice injected with CFA. Figure S2. Different dosages of FMNT had no analgesic effects in mice injected with CFA. (PDF 243 kb) [file 13041_2019_453_MOESM1_ESM.pdf]

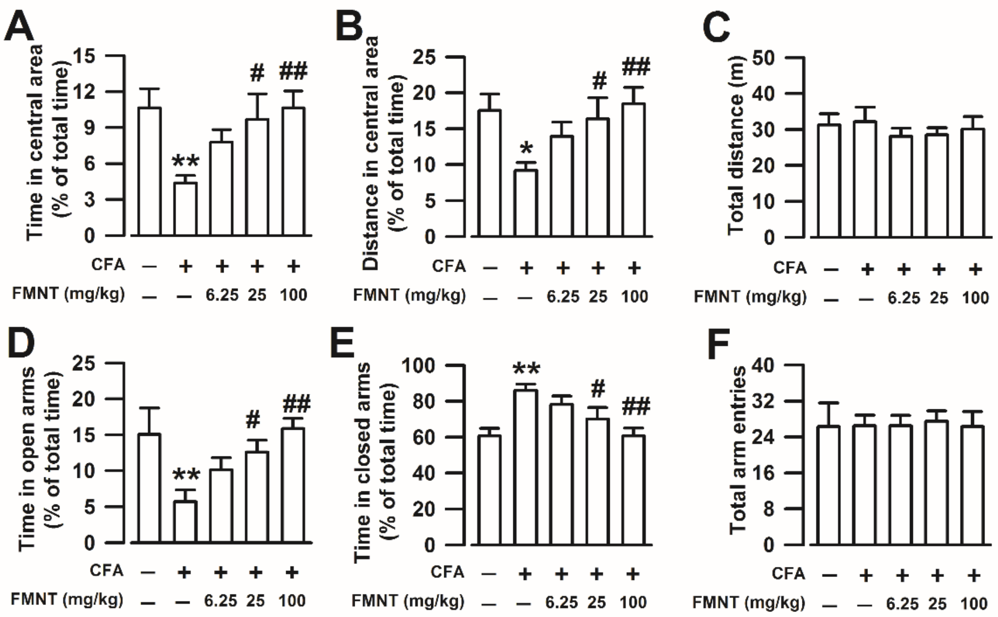

Supplementary Fig. 1 Anxiolytic effect of FMNT was dose-dependent in mice injected with CFA

Mice were divided into five groups: control group, CFA group, and other three groups administrated with different doses of FMNT after CFA injection. In OF test, treatment with FMNT (25 or 100 mg/kg) for 8 days significantly increased the time (**a**) and distance (**b**) spent in the central area. But there was no statistical difference between low dose group (6.25 mg/kg) and CFA-injected group. The total traveled distance was not affected (**c**). In EPM test, FMNT treatment increased the time spent in open arms (**d**) and decreased the time spent in closed arms (**e**) in a dose-dependent manner. The total arm entries in EPM test were comparable among these groups (**f**). n = 6 per group. $^{*}p < 0.05$, $^{**}p < 0.01$ vs. control; $^{\#}p < 0.05$, $^{\#\#}p < 0.01$ vs. CFA.

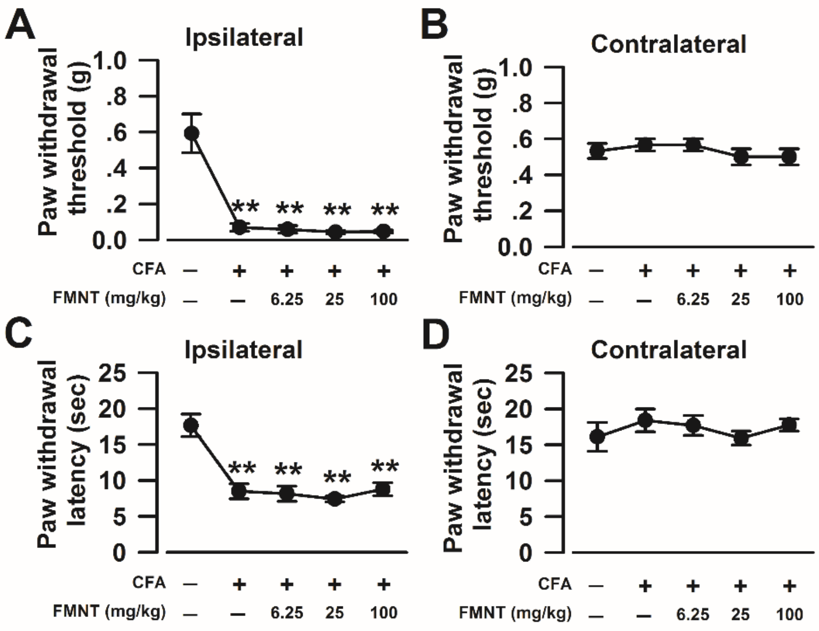

Supplementary Fig. 2 Different dosages of FMNT had no analgesic effects in mice injected with CFA

In Von Frey and hot plate tests, different dosages of FMNT (6.25, 25 or 100 mg/kg) did not alleviate CFA-induced mechanical allodynia (**a**) and thermal hyperalgesia (**c**) in CFA-injected hind paw (ipsilateral). But mechanical allodynia (**b**) and thermal hyperalgesia (**d**) in contralateral hind paw were not changed among these groups. n = 6 per group. **p < 0.01 vs. control.
